# Specialized Pro‐Resolving Mediators as Emerging Players in Cardioprotection: From Inflammation Resolution to Therapeutic Potential

**DOI:** 10.1111/apha.70062

**Published:** 2025-05-28

**Authors:** Anna De Bartolo, Naomi Romeo, Tommaso Angelone, Carmine Rocca

**Affiliations:** ^1^ Cellular and Molecular Cardiovascular Physiology and Pathophysiology Laboratory, Department of Biology, E. And E. S. (DiBEST) University of Calabria Cosenza Italy; ^2^ National Institute of Cardiovascular Research (INRC) Bologna Italy

**Keywords:** cardioprotection, inflammation, myocardial ischemia/reperfusion, pro‐resolving mediators, signal transduction cardiac function

## Abstract

**Aim:**

Timely myocardial reperfusion is essential for restoring blood flow to post‐ischemic tissue, thereby reducing cardiac injury and limiting infarct size. However, this process can paradoxically result in additional, irreversible myocardial damage, known as myocardial ischemia–reperfusion injury (MIRI). The goal of this review is to explore the role of specialized pro‐resolving mediators (SPMs) in atherosclerosis and MIRI, and to assess the therapeutic potential of targeting inflammation resolution in these cardiovascular conditions.

**Methods:**

This review summarizes current preclinical and clinical evidence on the involvement of SPMs in the pathogenesis of atherosclerosis and MIRI, acknowledging that several cellular and molecular aspects of their mechanisms of action remain to be fully elucidated.

**Results:**

MIRI is a complex phenomenon in which inflammation, initially triggered during ischemia and further amplified upon reperfusion, plays a central role in its pathogenesis. Various cellular and molecular players mediate the initial pro‐inflammatory response and the subsequent anti‐inflammatory reparative phase following acute myocardial infarction (AMI), contributing both to ischemia‐ and reperfusion‐induced damage as well as to the healing process. SPMs have emerged as key endogenous immunoresolvents with potent anti‐inflammatory, antioxidant, and pro‐resolving properties that contribute to limit excessive acute inflammation and promote tissue repair. While dysregulated SPM‐related signaling has been linked to various cardiovascular diseases (CVD), their precise role in AMI and MIRI remains incompletely understood.

**Conclusion:**

Targeting inflammation resolution may represent a promising therapeutic strategy for mitigating atheroprogression and addressing a complex condition such as MIRI.

**Abbreviations**
ACS: acute coronary syndrome
ALX/FPR2: formyl peptide receptor 2
AMI: acute myocardial infarction
ASCVD: atherosclerotic cardiovascular disease
ATP: adenosine triphosphate
ATS: auricular tragus stimulation
CABG: coronary artery bypass grafting
CAD: coronary artery disease
CRS: cardiorenal syndrome
CV: cardiovascular
CVD: cardiovascular disease
Cx43: connexin 43
CYP450: cytochrome P450
DAMPs: damage‐associated molecular patterns
DHA: docosahexaenoic acid
eNOS: endothelial nitric oxide synthase
EPA: eicosapentaenoic acid
ER: endoplasmic reticulum
FOXO1: forkhead box O1
H/R: hypoxia/reoxygenation
HEPE: 18‐hydroxy‐eicosapentaenoic acid
HF: heart failure
HO‐1: heme oxygenase‐1
hsCRP: high‐sensitivity C‐reactive protein
HUVECs: human umbilical vein endothelial cells
IHD: ischemic heart disease
IL: interleukin
LAD: left anterior descending
LDL: low‐density lipoprotein
LPS: lipopolysaccharide
LTB4: leukotriene B4
LV: left ventricular
LXA4: lipoxin A4
MACE: major adverse cardiovascular events
MaR1: maresin 1
MaR2: maresin‐2
MaR‐Ls: maresin‐like lipid mediators
MCTR: maresin conjugate in tissue regeneration
MI: myocardial infarction
MIRI: Myocardial ischemia/reperfusion injury
NETs: neutrophil extracellular traps
NF‐κB: nuclear factor kappa B
OCT: optical coherence tomography
ORP150: oxygen‐regulated protein
PCI: percutaneous coronary intervention
PD1: Protectin D1
PGE2: prostaglandin E2
PPARα: peroxisome proliferator‐activated receptors
pro‐BNP: pro‐brain natriuretic peptide
RIC: remote ischemic conditioning
ROS: reactive oxygen species
RvD1: resolvin D1
RvE1: resolvin E1
SCAD: stable coronary artery disease
SPMs: specialized pro‐resolving lipid mediators
SR: sarcoplasmic reticulum
STEMI: ST‐segment elevation myocardial infarction
TLR4: toll‐like receptor 4
TLRs: toll‐like receptors
Tn: troponin
TNF‐α: tumor necrosis factor‐alpha
TNF‐β: tumor necrosis factor‐beta
WBC: white blood cell

## 1 Background and Rationale

Atherosclerosis represents the primary underlying cause of cardiovascular disease (CVD), which continues to be the leading cause of death worldwide [1, 2, 3]. The accumulation of lipids, especially low‐density lipoprotein (LDL) cholesterol, within the arterial wall has historically been considered the primary risk factor for atherogenesis. However, since 1999, when atherosclerosis was recognized as an inflammatory disease [4], numerous signaling pathways, molecular antigens, and cell types involved in the inflammatory response have also been shown to contribute to the progression of atherogenesis. Atherogenesis is now understood as the result of a complex interplay between local and systemic inflammation, endothelial dysfunction, and active lipid accumulation [5, 6, 7]. As atherosclerosis advances, certain plaques develop a more unstable phenotype characterized by increased inflammation. This can trigger a complex cascade of events, ultimately leading to the clinical manifestation of myocardial infarction (MI) [8].

Myocardial ischemia/reperfusion injury (MIRI) is the pathophysiological substrate of ischemic heart disease (IHD), a manifestation of reduced coronary blood flow [9], and arises from an ischemic episode followed by the restoration of blood flow to post‐ischemic tissue. MIRI is a complex process involving multifactorial molecular and cellular interactions, where inflammation, initially triggered during ischemia, is further amplified upon reperfusion. This heightened inflammatory response contributes to infarct expansion and subsequent cardiac remodeling [10, 11]. The initial pro‐inflammatory response during MI, aimed at removing necrotic cell debris from the MI zone, is followed by an anti‐inflammatory reparative phase. Both phases are tightly regulated through complex interactions between cardiac and immune cells. Disruptions in the signaling pathways that govern the balance and transition between these pro‐inflammatory and anti‐inflammatory reparative processes lead to unresolved inflammation, contributing to MIRI‐associated pathological alterations [12]. Therefore, understanding the endogenous pro‐resolving processes is critical for identifying specific pathways that could serve as promising therapeutic targets. In particular, the resolution of inflammation is mediated by a group of lipid mediators known as specialized pro‐resolving lipid mediators (SPMs), which play key roles in reducing leukocyte infiltration and enhancing efferocytosis, leading to anti‐inflammatory and antioxidant effects [13].

This review aims to discuss the latest findings regarding the role of SPMs in MI and MIRI, recognizing that key mechanisms underlying their actions in these pathological conditions remain partially understood or, for certain SPMs, are still in the early stages of investigation. Additionally, it will highlight preclinical and clinical studies that suggest stimulating the resolution of inflammation may be beneficial for atheroprogression and for addressing a complex condition such as MIRI.

## 2 Brief Overview on MIRI

**FIGURE 1:**
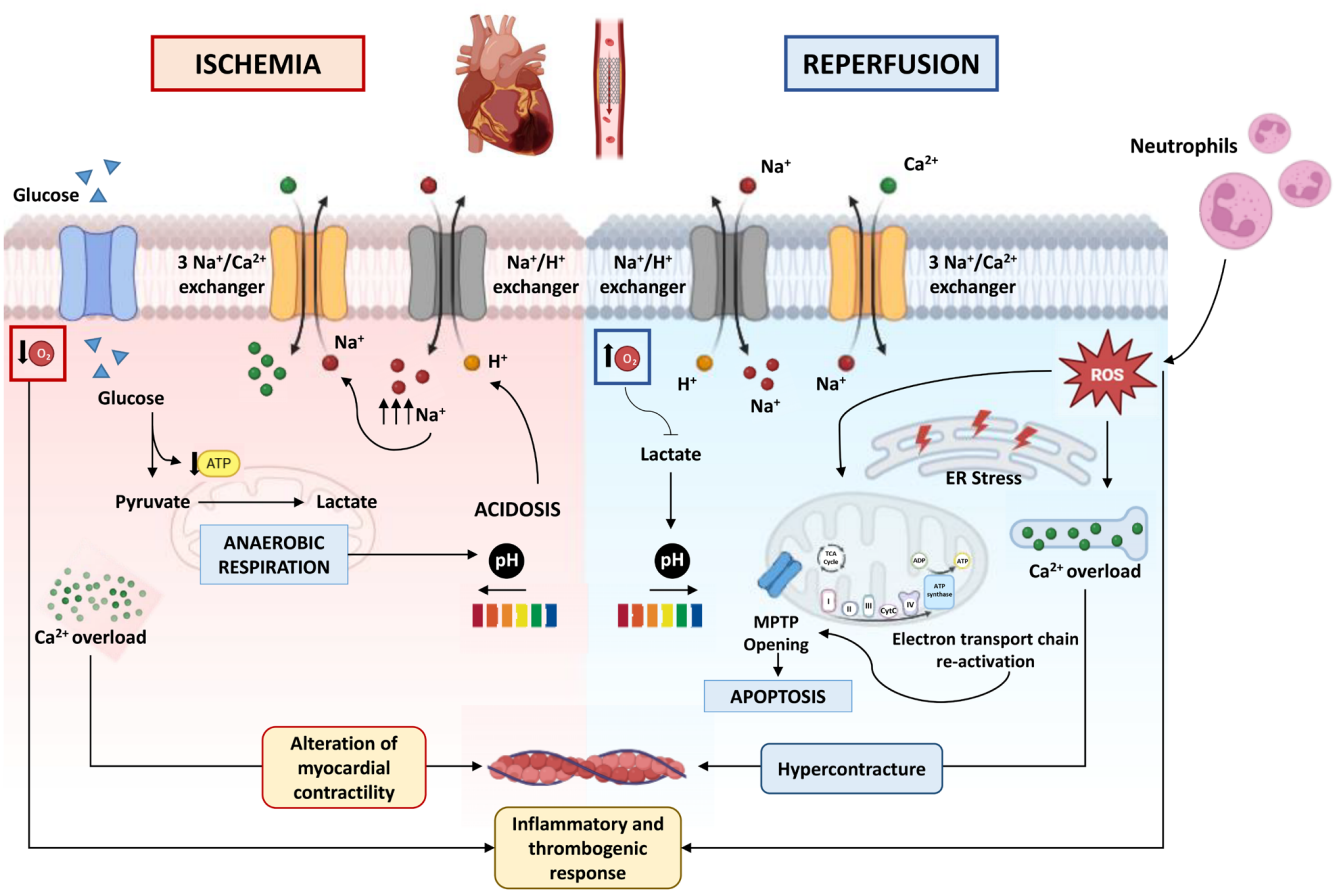
Mechanisms underlying myocardial ischemia/reperfusion injury (MIRI). The figure illustrates the key events associated with MIRI. During acute myocardial ischemia, oxygen deprivation forces cells to rely on anaerobic respiration, leading to lactate accumulation and a decrease in intracellular pH. Acidosis‐induced activation of the Na^+^/H^+^ exchanger results in Na^+^ overload, which in turn triggers the Na^+^/Ca^2+^ exchanger, causing an increase in intracellular Ca^2+^ levels. These alterations ultimately impair myocardial contractility. Upon reperfusion, oxygen availability is restored, normalizing intracellular pH and reestablishing the Na^+^/H^+^ and Na^+^/Ca^2+^ exchangers. However, excessive reactive oxygen species (ROS) production disrupts sarcoplasmic reticulum (SR) function, leading to hypercontracture, endoplasmic reticulum (ER) stress, and mitochondrial permeability transition pore (MPTP) opening, which induces cardiomyocyte apoptosis. Oxidative stress further amplifies inflammatory and thrombogenic responses, exacerbating myocardial injury.

As well established, in the context of reperfusion therapy for patients typically presenting with an acute ST‐segment elevation myocardial infarction (STEMI), early revascularization remains the most effective intervention to reduce cardiac injury and limit the extension of infarct size [14]. However, reperfusion by percutaneous coronary intervention (PCI) or surgical coronary artery bypass grafting (CABG) can paradoxically lead to accelerated and additional irreversible myocardial damage. Through multiple mechanisms, sustained MIRI promotes cardiomyocyte death in potentially salvable ischemic tissue and coronary microvascular injury, which ultimately leads to progressive myocardial metabolic, functional, and structural alterations contributing to heart failure (HF) and increased mortality and morbidity [15, 16] (Figure 1). The main pathophysiological events occurring in the acute MIRI are distinct across its two phases. During ischemia, alterations in cardiac metabolism depend on ischemia severity; in the case of absence of oxygen, energy metabolism is disrupted, the intracellular availability of the high energy phosphate, adenosine triphosphate (ATP), is reduced, and the cell shifts to anaerobic respiration, resulting in lactate accumulation and a subsequent decrease in intracellular pH. Intracellular acidosis during ischemia induces the activation of the Na^+^/H^+^ ion exchanger, which expels protons from the cell in exchange for Na^+^ entry. Moreover, ischemia attenuates the Na^+^ K^+^‐ATPase pump reducing Na^+^ extrusion. The resulting intracellular Na^+^ overload triggers the 3Na^+^‐Ca^2+^ exchanger to operate in reverse, extruding Na^+^ while causing intracellular Ca^2+^ overload, which alters myocardial contractility and can lead to cell death [17, 18] (Figure 1). During reperfusion, the myocardium undergoes severe biochemical and metabolic changes that exacerbate the alterations generated during ischemia. Rapid alterations in ion flux and renormalization of pH reestablish the activity of Na^+^/H^+^ exchange and Na^+^/Ca^2+^ exchange, causing intracellular Ca^2+^ overload and paradoxically aggravating cell damage. The re‐establishment of blood flow and increasing oxygen supply reactivate the electron transport chain, producing reactive oxygen species (ROS) (the “oxygen paradox”) and generating intracellular oxidative stress that induces cardiomyocyte death through the opening of the mitochondrial permeability transition pore (PTP) [19, 20]. Moreover, ROS impair sarcoplasmic reticulum (SR) function, exacerbating intracellular Ca^2+^ overload, which in turn induces endoplasmic reticulum (ER) stress and contributes to cardiomyocyte hypercontracture [21, 22]. ROS also damage macromolecules and initiate inflammatory and thrombogenic responses that aggravate cardiomyocyte injury [20]. Additional ROS production is induced by neutrophils mobilized during reperfusion through chemotaxis and endothelial adherence within the vascular space, alongside platelets and CD4^+^ T‐lymphocytes. Neutrophils, in turn, stimulate tumor necrosis factor‐alpha (TNF‐α), TNF‐beta (TNF‐β), interferon‐gamma, and macrophage‐stimulating factors, further exacerbating tissue damage [23] (Figure 1). Oxidative stress produced during MIRI induces left ventricular (LV) remodeling and compromises post‐ischemic myocardial recovery, making it a major driver of reperfusion injury [24, 25]. On the other hand, a complex interplay of several other mechanisms, including the exacerbation of necrosis, deregulation of apoptosis and autophagy, along with inflammatory cascades and abnormalities in mitochondrial quality control processes, has been proposed to mediate cardiomyocyte and coronary microvascular injury in MIRI pathophysiology [15, 26].

### 2.1 Central Role of Inflammation in MIRI

A substantial body of evidence indicates that sustained inflammation and immune response play a direct role in the initiation of ischemic myocardial injury and following its acute event, as well as in MIRI pathology [27]. While standard post‐revascularization therapies, such as beta‐blockers, ACE inhibitors, and statins, primarily aim to prevent subsequent MI episodes and adverse LV remodeling, they may exert indirect anti‐inflammatory effects; they do not directly target the acute inflammatory response [28, 29]. Therefore, targeting inflammatory pathways and inflammatory molecules generated during MIRI or in the early phase after AMI has gained clinical attention. In this context, numerous agents, including but not limited to colchicine, anakinra, interleukin (IL)‐6 and IL‐1β inhibitors, have shown efficacy in preclinical studies (often performed in animal models with isolated disease) and smaller‐scale trials, but further evaluation in larger patient cohorts is necessary [11, 30].

The acute and chronic inflammation are often underestimated cardiovascular (CV) risk factors. However, their harmful role in acute MI (AMI) is evident. Indeed, important evidence indicates that many AMI patients do not exhibit elevated LDL cholesterol levels, a major causal risk factor for AMI, but instead display heightened inflammation. In addition, post‐AMI patients more frequently present with residual inflammation rather than residual augmented LDL‐C [31, 32]. On the other hand, inflammation significantly increases during the reperfusion phase and plays a crucial role in the generation of AMI size and post‐MI adverse LV remodeling [25, 33].

The concept that inflammation is not an isolated phenomenon confined to the heart but is instead influenced by numerous processes including altered metabolic status and immune cell activation in the setting of AMI, along with other factors such as age, comorbidities, and events preceding acute MI, has led researchers to uncover multiple immunopathological mechanisms and inflammatory pathways and their complex interactions [11]. In this context, recent outstanding studies have summarized the key cellular and molecular events underlying the various phases of the inflammatory response during AMI (see [11, 12, 30, 32, 34, 35]). These studies also emphasized how functional changes and differing kinetics of major cell types (macrophages, neutrophils, and lymphocytes) during AMI orchestrate the inflammatory, proliferative, and maturation phases of healing, offering significant therapeutic implications.

**FIGURE 2:**
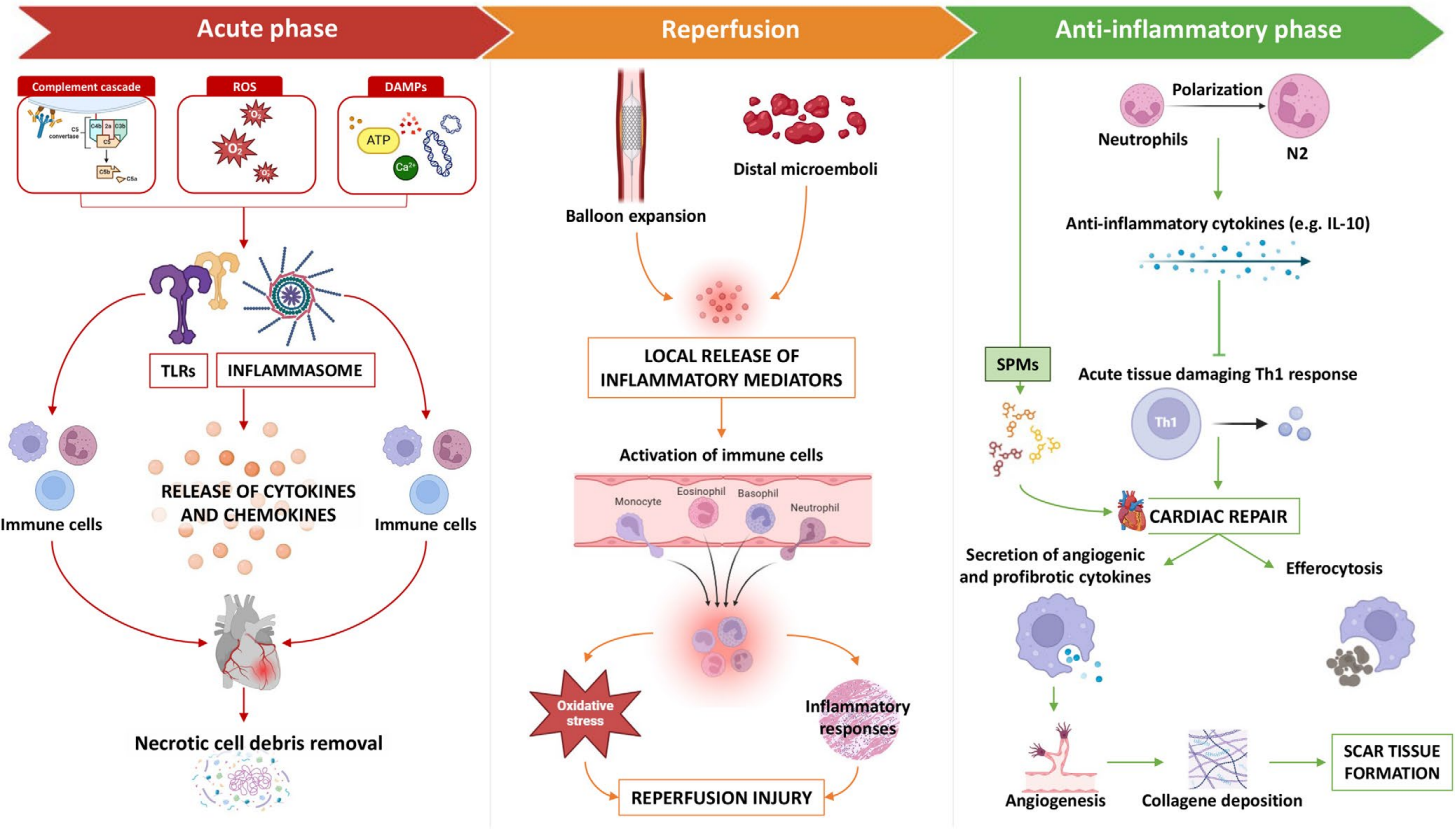
Overview of pro‐inflammatory and anti‐inflammatory responses in myocardial ischemia/reperfusion injury (MIRI). In the acute phase following myocardial infarction (MI), activation of the complement cascade, reactive oxygen species (ROS), and damage‐associated molecular patterns (DAMPs) stimulate toll‐like receptors (TLRs) and inflammasomes, leading to an increased release of cytokines and chemokines. This promotes the recruitment of immune cells to facilitate the clearance of necrotic cell debris. Following reperfusion, balloon and stent dilation, along with distal microembolization, further enhance the local release of inflammatory mediators. This process intensifies immune cell activation, amplifying oxidative stress and inflammatory responses. The initial pro‐inflammatory phase is subsequently followed by an anti‐inflammatory response, during which neutrophils polarize to a reparative phenotype and produce anti‐inflammatory cytokines that contribute to cardiac repair. Specialized pro‐resolving mediators (SPMs) also play a crucial role in this phase by promoting efferocytosis and reducing pro‐inflammatory signaling, supporting tissue healing and inflammation resolution.

During the acute phase of myocardial ischemia, an initial pro‐inflammatory response aims to remove necrotic cell debris from the MI zone (Figure 2). This process is mediated by complement cascade activation, ROS, and damage‐associated molecular patterns (DAMPs), such as extracellular calcium, mtDNA, and ATP released from the infarcted myocardium, that activate toll‐like receptors (TLRs) and inflammasomes to release pro‐inflammatory cytokines and chemokines, through which mediate the rapid recruitment from the circulation and accumulation of several immune cells into the infarct zone [6, 36]. Here, neutrophils polarize to an inflammatory phenotype, while monocytes differentiate into inflammatory macrophages, releasing inflammatory mediators, ROS, and neutrophil extracellular traps (NETs) to promote debris clearance [37]. In STEMI patients, early PCI provokes a local release of inflammatory mediators due to balloon and stent expansion, as well as distal microemboli, further recruiting immune cells, amplifying oxidative stress and the inflammatory response and causing reperfusion injury, which manifests 6–24 h after the reperfusion therapy [12, 32, 38] (Figure 2).

Following the pro‐inflammatory response, a post‐AMI anti‐inflammatory phase occurs on days 4–7 to repair inflammation [37]. This complex process, aimed at suppressing and resolving the initial pro‐inflammatory phase, is driven by the activation of endogenous inhibitory pathways that suppress the inflammatory phenotype in infiltrated leukocytes within the MI zone [39]. After the initial cardiac inflammatory resolution and repair, neutrophils polarize toward a reparative (“N2”) phenotype where they are required for the appropriate later wound healing phase; in particular, through multiple molecular processes accompanied by the production of anti‐inflammatory cytokines (e.g., IL‐10), they suppress the acute tissue‐damaging Th1 response and contribute to cardiac repair [40]. Following efferocytosis of apoptotic neutrophils and cardiomyocytes, macrophages shift to an anti‐inflammatory reparative phenotype and, by secreting anti‐inflammatory, angiogenic, and profibrotic cytokines, mitigate the further proinflammatory leukocyte influx and induce angiogenesis and collagen deposition to promote scar tissue formation [34] (Figure 2).

### 2.2 SPMs In MIRI

Among the mediators involved in resolving acute inflammation in AMI and MIRI, SPMs have emerged as potential key players. SPMs, including the lipoxin, resolvin, protectin, and maresin families, are a group of molecules biosynthesized from polyunsaturated fatty acids that exhibit potent anti‐inflammatory and pro‐resolving properties and play vital roles in host defense, pain regulation, organ protection, and tissue remodeling [41]. SPMs are locally produced at sites of tissue injury and do not merely act as passive inhibitors of inflammatory pathways. Rather, they actively regulate numerous intracellular mechanisms through high‐affinity interactions with specific receptors that induce cell‐type specific responses to initiate pro‐resolving programs, while limiting excessive leukocyte infiltration and pro‐inflammatory signaling, thereby contributing to the restoration of homeostasis [13, 42, 43]. SPMs also stimulate innate immune cells to eliminate microbes and support the resolution of inflammation and tissue repair. This is achieved by promoting monocyte recruitment and regulating macrophage activity, particularly through the enhancement of apoptotic cell clearance at inflammatory sites and the acceleration of lymphatic drainage [44].

In the specific context of AMI, SPMs activate specific and distinct cellular receptors to reduce pro‐inflammatory mediator production, modulate neutrophil phenotypes, promote neutrophil apoptosis, enhance macrophage‐mediated clearance of apoptotic neutrophils, and support reparative macrophage polarization [45, 46]. All these functions mediated by SPMs are suggestive of common downstream pathways required for resolution‐mediators and plaque stability, supporting their active role in advanced atherosclerosis [47]. Notably, a sustained inflammatory state resulting from disrupted inflammation‐resolution processes involving these mediators has been linked to the progression of numerous CVD including atherosclerotic vascular disease, ultimately leading to AMI [47]. Accordingly, pre‐clinical evidence underscores the fundamental and emerging role for SPMs in the heart and vasculature following revascularization, thus supporting their active role in the initiation and resolution of MIRI.

#### 2.2.1 The Immunomodulatory Role of the Spleen in Inflammation and Resolution in Coronary Atherosclerosis and Reperfused Acute Myocardial Infarction

In addition to macrophages, SPMs are also produced by, and act upon, other components of the adaptive immune system, including B cells and T cells. These mediators have been detected in all sites involved in B cell development, such as the bone marrow, peripheral blood, spleen, and lymph nodes, indicating that SPMs can spatiotemporally affect B‐cell function in vivo [48, 49]. The spleen, in particular, represents a major source of SPMs formation and a reservoir of immune cells, including neutrophils, monocytes, macrophages, B cells, and T cells, that can infiltrate atherosclerotic plaques and the infarcted myocardium, thereby influencing plaque progression and destabilization. As a central regulator of lipid metabolism and a key immune organ that also contributes to extramedullary hematopoiesis, the spleen has emerged as a crucial player in both the inflammatory and reparative phases of AMI and in post‐ischemic cardiac remodeling [50, 51, 52]. In mice subjected to coronary ligation leading to irreversible HF, leukocytes have been shown to mobilize from the spleen to the infarcted heart, where they contribute to the production of SPMs that promote myocardial healing and recovery [53]. Notably, higher levels of SPMs in the spleen compared to the LV prior to MI, along with a marked increase in SPMs within the infarcted LV within 24 h post‐MI, were found to temporally correlate with the resolution of inflammation [53]. Complementing these findings, Swirski et al. [54] demonstrated that splenic monocytes are rapidly mobilized to the infarcted myocardium within 24 h following ischemic injury, where they contribute to wound healing processes.

However, the role of the spleen in atherosclerosis and MI is multifaceted. This complexity is highlighted by the differing outcomes of splenectomy performed before versus after coronary occlusion in experimental models, as well as by the different effects of specific spleen‐derived B and T cell subtypes, which can either promote or mitigate atherosclerosis and differentially contribute to myocardial injury or protection [50]. On the other hand, the role of spleen‐derived platelets in the inflammatory response during MI and in the early reperfusion phase remains to be fully elucidated, and their potential contribution should not be underestimated [55, 56].

The spleen–heart axis in MI is further highlighted by recent evidence in pigs and rats demonstrating a cardioprotective effect dependent on splenic activation through vagal nerves during remote ischemic conditioning (RIC) [57]. This effect is mediated by the release of humoral cardioprotective factors from the spleen, ultimately leading to a reduction in infarct size. Notably, these findings have been corroborated in human studies, which identified a direct role for vagal innervation and the spleen as a key relay organ in the signal transduction pathway underlying the cardioprotective effects of both RIC and non‐invasive auricular tragus stimulation (ATS), a procedure that has been associated with reduced release of cardiac injury biomarkers, fewer ventricular arrhythmias, and improved contractile function in patients with AMI [58].

These findings highlight the existence of a complex and dynamic spleen–heart cross‐talk that regulates leukocyte trafficking and SPM biosynthesis in the infarcted myocardium and spleen following MI, ultimately determining the intricate balance between inflammatory and resolving responses.

#### 2.2.2 Lipoxin A4 (LXA4)

**FIGURE 3:**
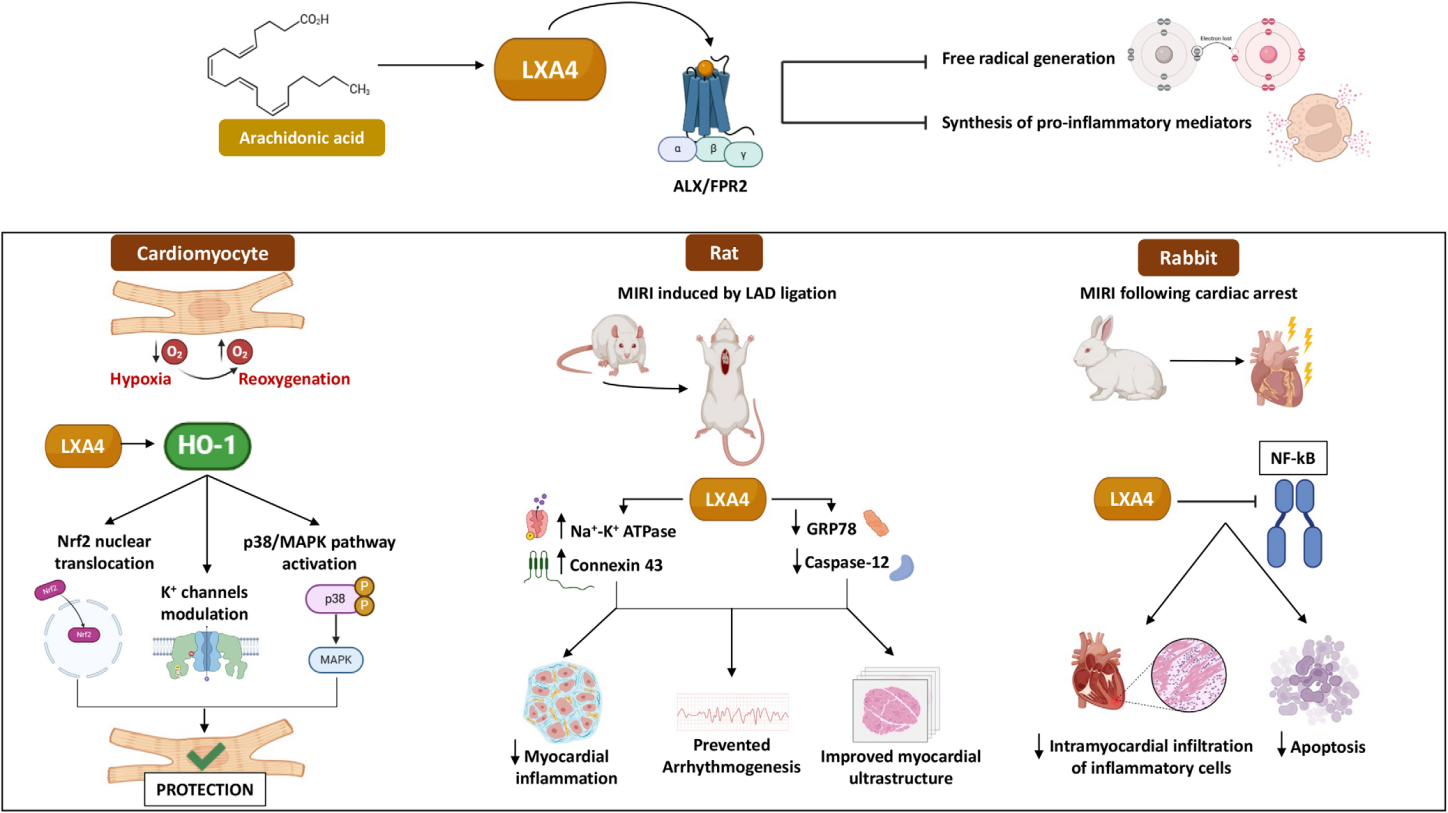
Implications of Lipoxin A4 (LXA4) in mitigating myocardial ischemia/reperfusion injury (MIRI): Translational evidence. The figure illustrates the cardioprotective effects of LXA4 demonstrated in both in vitro and in vivo studies. LXA4 is synthesized from arachidonic acid and exerts potent anti‐inflammatory and antioxidant effects by binding to the G‐protein–coupled receptor ALX/FPR2, thereby inhibiting free radical generation and the synthesis of pro‐inflammatory mediators. In vitro, LXA4 protects cardiomyocytes from hypoxia/reoxygenation (H/R) injury by upregulating heme oxygenase‐1 (HO‐1), activating the p38/MAPK pathway, modulating K^+^ channels, and promoting the nuclear translocation of Nrf2. In vivo, LXA4 confers cardioprotection in rat models of left anterior descending (LAD) coronary artery ligation‐induced ischemia followed by reperfusion, reducing myocardial inflammation and preventing arrhythmogenesis. Additionally, LXA4 has been shown to preserve myocardial ultrastructure by activating Na^+^‐K^+^ ATPase and Connexin 43 while inhibiting GRP78 and caspase‐12. In rabbit models of myocardial ischemia–reperfusion injury (MIRI), LXA4 suppresses NF‐κB activation, thereby mitigating intramyocardial inflammatory cell infiltration and apoptosis.

LXA4 is a small anti‐inflammatory lipid molecule derived from arachidonic acid, whose direct pro‐resolving and anti‐inflammatory actions have been demonstrated through pharmacological and loss‐of‐function approaches. These studies indicated that LXA4 exerts its effects by interacting with formyl peptide receptor 2 (ALX/FPR2), a multifunctional G‐protein‐coupled receptor that binds various ligands, thereby mediating either the initiation or resolution of inflammation [59, 60]. Given the potent inhibitory action of LXA4 on the synthesis of pro‐inflammatory mediators and free radical generation, there is reasonable evidence to suggest that its deficiency may perpetuate inflammation and contribute to different CVD, including MI and diabetes‐associated atherosclerosis [61, 62]. Over the past years, both in vitro and in vivo studies have also indicated that LXA4/LXA4 receptor activation can afford cardioprotection against MIRI. Based on the assumption that LXA4 protects H9c2 cardiomyocytes from hypoxia/reoxygenation (H/R) injury by upregulating heme oxygenase‐1 (HO‐1) to activate the p38 MAPK pathway, promote the nuclear translocation of Nrf2, and modulate ATP‐sensitive K^+^ channels as well as calcium‐sensitive K^+^ channels [63, 64], Zhao et al. [65] provided in vivo evidence of LX4A cardioprotective effects in a rat model of MIRI induced by left anterior descending (LAD) coronary artery ligation followed by reperfusion. Specifically, the authors found that LX4A infusion alleviates myocardial inflammation and oxidative stress while upregulating Na^+^‐K^+^‐ATPase and connexin 43 (Cx43) to improve myocardial ultrastructure and prevent arrhythmogenesis following MIRI [65] (Figure 3). Similar findings demonstrated the ability of LX4A to downregulate GRP‐78 and caspase‐12, thereby inhibiting neutrophil activation and attenuating myocardial inflammatory and oxidative stress, ultimately inducing myocardial protection in a rat model of MIRI [66]. The beneficial effects of LXA4 were further confirmed in a rabbit model of MIRI following cardiac arrest, demonstrating its ability to suppress nuclear factor kappa B (NF‐κB) activation, reduce intramyocardial infiltration of inflammatory cells, and mitigate myocardial apoptosis [67] (Figure 3). Interestingly, the peptide CR‐AnxA12‐48, which exhibits high affinity and specificity for LXA4, as well as strong resistance to neutrophil‐mediated degradation, has been shown to reduce infarct size and systemic chemokine (C‐C motif) ligand 5 concentrations following MIRI. By acting as an LXA4 agonist in mice, it protects the heart from leukocyte‐mediated tissue damage [68]. More recently, the role of the stable LXA4 form, 15‐epimer LXA4 (15‐epi‐LXA4), in the post‐MI acute inflammatory response and resolution phase has been investigated [69]. The authors found that both 15‐epi‐LXA4 with liposomal fusion (Lipo‐15‐epi‐LXA4) and free 15‐epi‐LXA4 effectively initiated the resolution phase in post‐MI healing, limiting cardiac remodeling and subsequent HF. This effect was mediated not only through agonism of FPR2 and GPR120 but also through antagonism of GPR40. Notably, Lipo‐15‐epi‐LXA4 also exerted anti‐inflammatory effects in the remote organ kidney, highlighting its high therapeutic potential in mitigating cardiac remodeling and delaying cardiorenal failure post‐MI.

In addition to these findings emphasizing the direct cardioprotective role of LX4A in preclinical MIRI settings, other studies have indicated that the cardioprotective profile of numerous pharmacological agents against MIRI is associated with increased levels of LX4A. This is true for ticagrelor, alone or in combination with rosuvastatin, whose cardioprotective action depended on adenosine receptor–mediated activation of Akt, eNOS, and COX‐2, resulting in increased production of prostacyclin and 15‐epi‐LXA4, or enhanced COX‐2 activity and myocardial levels of 6‐Keto‐PGF1α and 15‐epi‐LXA4 [70, 71]. Clinical reports clearly supported these findings. In particular, a prospective cohort study that recruited 1569 AMI patients reported a significant correlation between high circulating levels of LXA4 (≥ 5.637 ng/mL) and a lower risk of recurrent ischemic events in AMI patients [72]. Moreover, high LXA4 levels, together with low levels of high‐sensitivity C‐reactive protein [hsCRP (< 5.7 mg/L)], were associated with a lower risk of major adverse cardiovascular events (MACE), suggesting a significant prognostic impact of LXA4 in atherosclerotic individuals [72]. Similarly, a single‐arm prospective trial involving 48 individuals with coronary artery disease (CAD) showed that only patients who experienced an improvement in myocardial ischemia and failure following extracorporeal shockwave myocardial revascularization (ESMR) exhibited increased markers of angiogenesis (i.e., VEGF), decreased markers of inflammation (i.e., IL‐1β), and elevated levels of pro‐resolving mediators (i.e., LXA4) [73]. A very recent study based on 81 patients with MI reported that the LXA4 level per CRP was significantly lower in MI patients than in healthy subjects [74]. Additionally, LXA4 levels in MI subjects positively correlated with the percentage of docosahexaenoic acid (DHA) and negatively correlated with the number of stents implanted. Although further studies involving a larger number of patients are necessary, these findings suggest that LXA4 levels and the LXA4/CRP ratio may serve as valuable additional clinical markers for improving the diagnosis and management of the atherosclerotic processes driving CAD.

#### 2.2.3 Resolvin D1 (RvD1)

**FIGURE 4:**
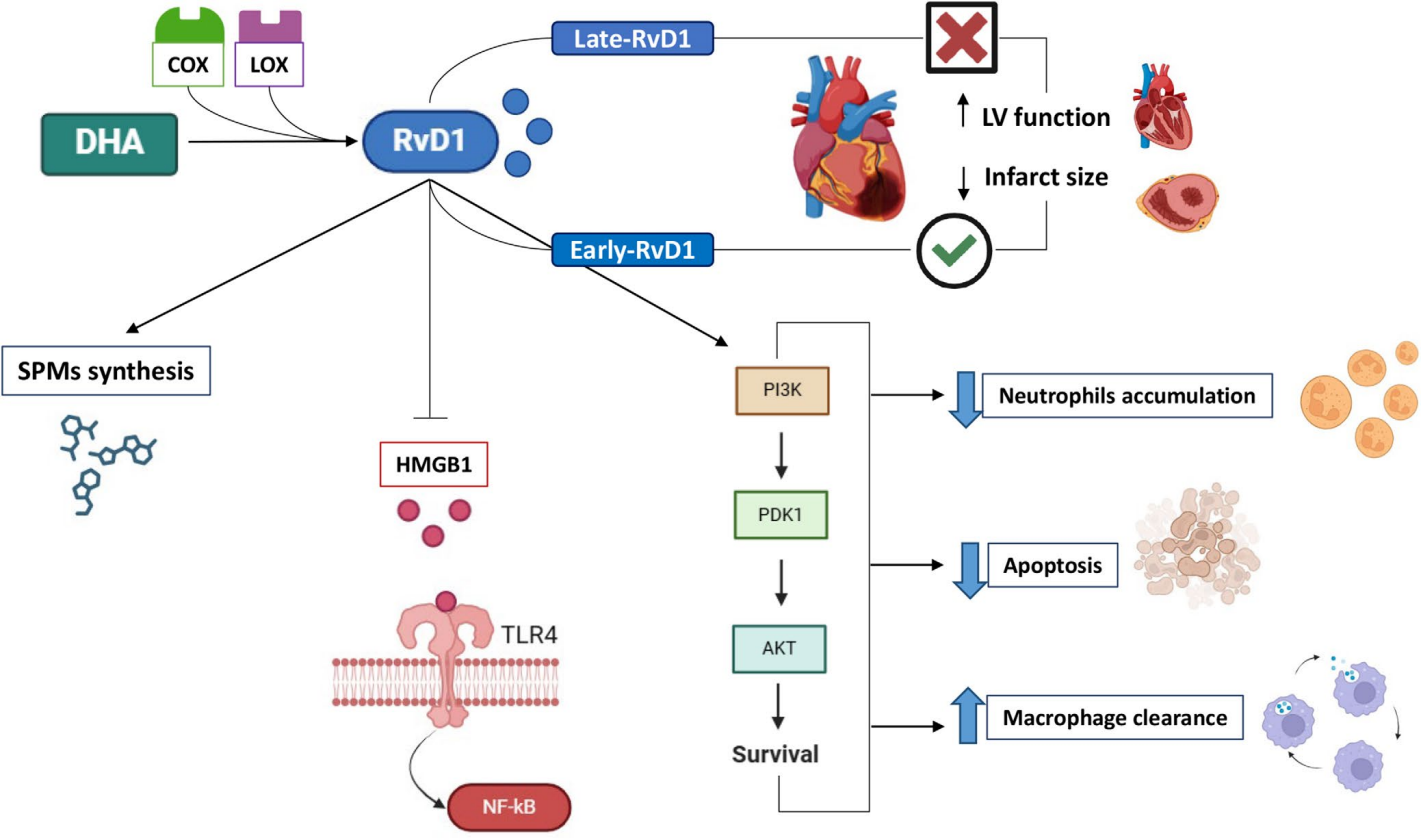
Potential cardioprotective effects of Resolvin D1 (RvD1) against myocardial ischemia/reperfusion injury (MIRI). This schematic representation outlines the potential mechanisms through which RvD1, a lipid mediator derived from docosahexaenoic acid (DHA), exerts cardioprotection. Early administration of RvD1, but not late administration, has been shown to reduce infarct size and improve left ventricular (LV) function. On the other hand, RvD1 promotes inflammation resolution by enhancing the synthesis of other specialized pro‐resolving mediators (SPMs) and downregulating high‐mobility group box‐1 (HMGB1) and its downstream signaling pathways, including TLR4 and NF‐κB. Moreover, RvD1 activates the pro‐survival PI3K/Akt pathway, which helps restore macrophage‐mediated clearance, reduces neutrophil accumulation in infarcted areas, and limits cell death, thereby contributing to improved cardiac recovery.

RvD1 is a member of the resolvin family, a subgroup of SPMs derived from DHA; it exerts antioxidant action and plays a direct role in rebalancing pro‐ and anti‐inflammatory lipid mediators by controlling neutrophil priming and reprogramming inflammatory macrophages toward the healing response. Moreover, RvD1 can promote the synthesis of other SPMs, thereby generating favorable feedback that promotes inflammation resolution [75]. This contributes to the stabilization of vulnerable plaques and to the inhibition of atherosclerosis progression [76, 77]. Mass spectrometry imaging analyses revealed the presence of augmented bioactive lipid resolving molecules, including RvD1, in the infarcted LV compared with no‐MI controls, indicating that the infarcted myocardium serves as the primary site for inflammation‐resolution pathomechanics, which are crucial for resolving inflammation [78]. Accordingly, the beneficial effects of RvD1 in improving myocardial function following MI and cardiac arrest have been observed in numerous studies highlighting its potential therapeutic value in cardioprotection and post‐MI cardiac healing. For instance, the cardioprotective effect of RvD1 was tested in a LAD ligation mouse model, where both the free acid form of RvD1 and RvD1 incorporated into liposomes (Lipo‐RvD1) were infused for 1 day or up to the fifth day post‐MI [79]. The results indicated that RvD1 significantly attenuates neutrophil recruitment in the spleen and LV, enhances resolving lipid mediators, and stimulates macrophage clearance in the post‐ischemic phase, while stabilizing the ECM by regulating LOX and COX enzymes. These actions, in turn, promote the resolution of inflammation, thereby mitigating the progression of post‐MI LV dysfunction [79] (Figure 4). On the other hand, one intraventricular injection of RvD1 before coronary occlusion protected rats from MIRI‐induced neutrophil accumulation and apoptosis, thus limiting the extension of the infarct size through the phosphoinositide 3‐kinase (PI3K)/Akt signaling pathway [80]. The same research group further explored the mechanisms of action underlying RvD1 cardioprotection following MIRI, reporting that the inhibition of two enzymes involved in the formation of RvD1 from omega‐3 PUFA (i.e., COX‐2 and 15‐LOX) attenuates its cardioprotective effect. However, when COX‐2 and 15‐LOX were inhibited, RvD1 restored cardioprotection indicating the key role of RvD1 in omega‐3 PUFA‐dependent protection in MI [81]. In parallel, the omega‐6 PUFA linoleic acid was found to attenuate the cardioprotective action of RvD1 during MIRI, suggesting that a diet high in omega‐6 fatty acids may compromise the beneficial anti‐inflammatory and inflammation‐resolving effects of omega‐3 fatty acids. Therefore, controlling omega‐6 intake should be considered to preserve the beneficial action of RvD1, particularly in high‐risk CV patients [82]. Based on this data, intra‐myocardial RvD1 injection administered 5 min before 45 min of myocardial ischemia followed by 180 min of reperfusion attenuated myocardial injury, oxidative stress, and infarct size [83]. These effects were achieved by RvD1 due to its ability to mitigate high‐mobility group box‐1 (HMGB1) activation and its downstream signaling via NF‐κB and toll‐like receptor 4 (TLR4), major mediators of inflammation and MIRI [84] (Figure 4). The involvement of TLR4 in RvD1's myocardial action was recently corroborated by a study showing that the anti‐inflammatory‐mediated cardioprotection of the selective human TLR4 antagonist aptamer (4FT) in a rat model of MIRI was accompanied by enhanced RvD1 serum levels, that are required for a proper resolution of inflammation [85].

Research has reinforced the translational impact of RvD1 by testing its cardioprotective role on cardiac and cerebral outcomes after cardiopulmonary resuscitation (CPR) in a porcine model. Intravenous administration of both high and low doses of RvD1 dose‐dependently improved post‐resuscitation cardiac and cerebral outcomes by significantly reducing myocardial and cerebral inflammation, as well as oxidative stress [86]. It is worth mentioning that RvD1 may also be effective in cardiorenal syndrome (CRS) after acute MI‐induced HF in mice [87]. The authors indicated that RvD1 stimulates clearance of leukocytes from infarcted areas and macrophage plasticity toward a reparative phenotype while amplifying kidney reparative cytokines and reducing acute kidney inflammation. These findings suggest that RvD1 has the potential to mitigate progressive inflammation caused by the collateral damage of non‐cleared leukocytes, which are responsible for CRS. On the other hand, in a highly translational study, Hiram et al. (2024) [88] recently showed the active role of RvD1 in resolving inflammation during the development of atrial arrhythmogenic remodeling resulting from LV dysfunction induced by MI in rats. Importantly, while early‐RvD1 (i.e., before MI) improved LV function, decreased infarct size, and prevented atrial cardiomyopathy, late‐RvD1 (i.e., day 7 post‐MI) did not positively affect infarct size or LV dysfunction. However, similar to early treatment, it exerted anti‐inflammatory effects and enhanced pro‐resolution signaling (Figure 4). These findings not only highlight the therapeutic potential of RvD1 in the development and progression of atrial arrhythmogenic substrates but also emphasize the importance of considering the time‐dependent effects of SPMs supplementation following myocardial injury [89].

Although RvD1 exhibits cardioprotective actions against MI and MIRI, its biological instability and lack of targeted delivery may hinder its clinical translational potential [90]. To address this limitation, several recent studies utilizing diverse experimental models—ranging from autoimmune encephalomyelitis to osteoarthritis and lung infection [91, 92, 93, 94]—have focused on developing more stable analogs with improved pharmacokinetic and pharmacodynamic properties and novel targeted delivery systems with controlled release. These advancements aim to enhance RvD1's chemical stability while preserving its ability to resolve inflammation and exert antioxidant actions in vivo. Notably, the intravenous injection of a platelet‐bionic, ROS‐responsive RvD1 delivery platform has recently been shown to protect mice from MIRI [95]. This formulation was able to selectively deliver RvD1 to the injury site in response to high local ROS levels, facilitating the clearance of dead cardiomyocytes, promoting the production of SPMs, and ultimately improving cardiac function. However, further research is needed to develop strategies to enhance RvD1's metabolic stability in heart disease, including MIRI. Taking advantage of the cardioprotective action of RvD1 in experimental settings, a few clinical studies have recently tested whether MI affects RvD1 circulating levels. Accordingly, serum levels of RvD1 have been shown to be significantly lower in patients with STEMI compared to healthy subjects [96]. Moreover, RvD1 levels were negatively correlated with inflammatory markers [total white blood cell (WBC) count, neutrophil count, and hsCRP], thrombolysis in myocardial infarction (TIMI) thrombus grade, pro‐brain natriuretic peptide (pro‐BNP), and peak troponin (Tn) I level. Conversely, RvD1 levels were positively correlated with LV ejection fraction, and its levels at admission were associated with poor prognostic markers of STEMI [96]. Similarly, patients with acute coronary syndrome (ACS) exhibited higher levels of leukotriene B4 (LTB4), along with lower RvD1 levels and a reduced RvD1‐to‐LTB4 ratio compared to those with stable coronary artery disease (SCAD) [97]. These studies further support the significant role of RvD1 in systemic inflammation during human MI and suggest that its levels could serve as an independent predictor of STEMI. Moreover, the imbalance between SPMs and proinflammatory leukotrienes may provide insights into atherosclerotic plaque instability, thereby serving as a potential biomarker for ACS risk. In contrast, a clinical study involving 240 STEMI patients undergoing optical coherence tomography (OCT) examination reported that high levels of RvD1 were associated with plaque rupture, calcification, and healing of culprit lesions [98]. This may reflect unresolved arterial inflammation, which continuously recruits inflammatory cells to expanding plaques, thereby stimulating RvD1synthesis. Notably, circulating levels of SPMs, including those from the resolvin family, are highly modulated at the onset of STEMI, reaching their peak within hours of MI symptom onset, even before the observed maximum release of high‐sensitivity cardiac troponin T (hsTnT). This increase in SPMs is accompanied by a significant decline in pro‐inflammatory prostaglandins and thromboxane B2, suggesting that the resolution of inflammation during MI is an active, tightly regulated, and early process [99]. Additional studies are warranted to further elucidate the pro‐resolving antioxidant action of RvD1 in human atherosclerosis, as well as to determine how alterations in RvD1 influence chronic inflammation and impaired resolution within coronary atherosclerotic plaques.

#### 2.2.4 Resolvin E1 (RvE1)

RvE1 was first identified as a bioactive lipid mediator through the analysis of inflammatory exudates from mice treated with omega‐3 polyunsaturated fatty acids and aspirin [100]. It is specifically produced by eicosapentaenoic acid (EPA) metabolism via COX‐2 or CYP450 (cytochrome P450) pathway [101]. Accumulating evidence suggests RvE1 confers cardioprotection against lipopolysaccharide (LPS)‐induced heart injury [102], doxorubicin‐induced cardiotoxicity [103], and hypertension and vascular remodeling [104], all of which are widely prevalent CV conditions [105, 106, 107, 108]. The reduced or absent levels of specific SPMs, including RvE1, found in CAD patients compared to their presence in healthy patients, have been correlated with a diminished capacity to resolve acute inflammation, contributing to the progression of atherosclerosis [109, 110]. Although the role of RvE1 in MI has not been extensively explored, it has been identified as a key player in resolving inflammation triggered by ischemic and reperfusion injury. In this regard, an in vivo study has demonstrated that intravenously administered RvE1, given before reperfusion, dose‐dependently reduced infarct size in an open‐chest rat model of MIRI by preventing leukocyte infiltration into the post‐ischemic zone [111]. The direct cardioprotective effects of RvE1 were further confirmed in vitro, where RvE1 administration promoted endothelial nitric oxide synthase (eNOS) phosphorylation and activated pro‐survival PI3K/Akt and ERK1/2 pathways while mitigating pro‐apoptotic signaling [111]. Moreover, the study suggested that RvE1's cytoprotective action may also depend on epidermal growth factor receptor (EGFR) transactivation, as EGFR blockade abolished RvE1‐induced cardioprotection both in vivo and in vitro. However, the precise mechanisms by which RvE1 activates myocardial EGFR and the exact role of EGFR in MIRI remain to be fully elucidated. In a LAD‐induced MI mouse model, Liu et al. [112] investigated the effects of RvE1 administration at different time points on post‐ischemic myocardial recovery. Interestingly, RvE1 given during the first 7 days post‐MI (i.e., the acute phase of MI), but not when administered between days 7–14, significantly improved myocardial function. This cardioprotective effect was achieved by inhibiting the recruitment of the subpopulations of monocytes/macrophages Ly6C^hi^ Mo/Mp and reducing the secretion of pro‐inflammatory cytokines, thereby suppressing cardiomyocyte apoptosis [112]. This data suggests that RvE1 can serve as an early cardioprotective agent in MI, emphasizing the importance of timely intervention in its therapeutic application. However, a more comprehensive understanding of its long‐term effects and the molecular mechanisms through which RvE1 modulates pro‐survival kinases, inhibits pro‐apoptotic factors, and promotes the resolution of inflammation for cardiac repair after ischemia is still required.

#### 2.2.5 Maresin 1 (MaR1)

Together with maresin‐2 (MaR2), maresin conjugate in tissue regeneration (MCTRs), and maresin‐like lipid mediators (MaR‐Ls), MaR1 is a member of the family of “macrophage mediators in resolving inflammation” (maresins). Discovered in 2009 as potent anti‐inflammatory and proresolving mediators, maresins have a key role in wound healing, tissue regeneration and organ protection [113]. Based on this, numerous pre‐clinical studies have highlighted the therapeutic potential of MaR1 in inflammation‐related CVD [114]. However, the precise cellular and molecular mechanisms underlying its beneficial effects in cardiovascular tissue remain to be fully elucidated. Additionally, although further in vivo evidence is needed to establish the cardioprotective role of MaR1 against MI and MIRI, consistent findings indicate its key involvement in mitigating atherosclerosis progression by directly promoting macrophage polarization toward an M2‐like phenotype, enhancing efferocytosis, and facilitating tissue regeneration [115]. At cellular level, the effect of MaR1 on atherosclerotic response has been investigated in human umbilical vein endothelial cells (HUVECs) exposed to LPS insult [116]. MaR1 effectively alleviated endothelial pro‐inflammatory reaction through stimulating peroxisome proliferator‐activated receptors (PPARα) signaling, while simultaneously reducing ER stress. This was achieved through the upregulation of the 150 kDa oxygen‐regulated protein (ORP150) via a PPARα/forkhead box O1 (FOXO1)‐dependent pathway. An in vivo study explored the role and potential mechanisms of Mar1 in a mouse model of LAD‐induced MI. The study demonstrated that MaR1 effectively attenuated post‐MI oxidative stress and inflammation by activating the Nrf2/HO‐1 signaling pathway while inhibiting TLR4/NF‐κB signaling. These effects contributed to the suppression of myocardial apoptosis and interstitial fibrosis, reduced susceptibility to ventricular arrhythmias, and improved post‐ischemic myocardial recovery [117]. Consistent with this, in the aortas of atherosclerosis‐prone apolipoprotein E‐deficient (Apoe^−/−^) mice exposed to a high‐fat diet, increased levels of the inflammatory lipid mediators LTB4 and prostaglandin E2 (PGE2) were associated with decreased levels of MaR1 and resolving D2 (RvD2) during atheroprogression [118]. Notably, the administration of MaR1 and RvD2 prevented necrotic core expansion, reduced macrophages accumulation, and induced a reparative macrophage phenotype, thereby promoting plaque stability [118]. Similar conclusions were drawn from a 30‐month follow‐up clinical data conducted in 31 statin‐treated patients with stable CAD randomized based on higher or lower plasma levels of EPA + DHA [119]. The study examined correlations between the levels of RvE1, MaR1, and proinflammatory mediators (LTB4 and prostaglandins) with coronary plaque progression and regression evaluated by coronary computed tomographic angiography. The results indicated that subjects with higher plasma levels of EPA + DHA associated with increased levels of RvE1, MaR1, and RvE1 precursor 18‐hydroxy‐eicosapentaenoic acid (HEPE). Conversely, those with lower plasma EPA + DHA levels exhibited low (18‐HEPE+resolvin E1)/LTB4 ratio, which was associated with plaque progression [119]. A recent Chinese community‐based cohort study further supports the significant correlation between MaR1 circulating levels and the risk of atherosclerotic CVD (ASCVD), with this association being partially mediated by serum small dense low‐density lipoprotein cholesterol (sdLDL‐c) [120]. Among 2822 non‐ASCVD participants followed for 8 years, the study identified 290 new ASCVD cases and observed a linear dose–response relationship between MaR1 levels and incident ASCVD. Moreover, higher baseline plasma MaR1 levels were strongly associated with a lower risk of developing ASCVD [120].

Collectively, these studies strongly suggest that an imbalance in key lipid mediators can drive atherosclerosis progression, highlighting the administration of deficient SPMs or strategies able to increase SPMs or the SPM/LTB4 ratio as promising therapeutic approaches in atherosclerosis.

#### 2.2.6 Protectin D1 (PD1)

PD1 is an omega‐3 fatty acid synthesized from DHA, with well‐documented anti‐inflammatory and antioxidant effects in various in vitro and in vivo models of lung diseases, neurodegeneration, and ischemic stroke [121, 122, 123]. PD1 has also been shown to attenuate atherosclerosis progression by counteracting inflammatory cell infiltration and modulating the local inflammatory response, thereby reducing subsequent neointimal hyperplasia [124, 125]. However, the precise underlying mechanisms of PD1 in MI and MIRI are still in the early stages of investigation. Only a few studies have explored whether PD1 could also protect the reperfused heart. Recent findings suggest that, similar to its effects in the nervous system, PD1 alleviates oxidative stress and inflammatory responses, exerting myocardial protection in a rat model of LAD‐dependent MI [126]. Mechanistically, its cardioprotective action was mediated by the upregulation of miRNA‐210, a hypoxia‐induced microRNA known for its anti‐apoptotic function and role in vascular regeneration [127]. Additionally, PD1 activated the Akt/HIF‐1α signaling axis, which further suppressed apoptosis, highlighting a potential therapeutic mechanism for myocardial protection following MIRI.

## 3 Conclusions

It is now well established that inflammation plays a pivotal role in the pathogenesis of various CVD and is a key factor in MI and reperfusion injury. Disruptions in the intricate mechanisms governing the balance and transition between the pro‐inflammatory response triggered by MI and the subsequent anti‐inflammatory reparative process can lead to unresolved inflammation.

The increasing structural and functional characterization of endogenous anti‐inflammatory and pro‐resolving mediators highlights that inflammation resolution during MI is a complex, active, and tightly regulated process that influences infarct size and post‐MI LV remodeling. In this context, SPMs have emerged as crucial players, contributing to inflammation resolution by reducing the production of pro‐inflammatory mediators, limiting neutrophil infiltration, promoting the clearance of apoptotic neutrophils and cellular debris, and modulating macrophage phenotype transitions. These pro‐repair and pro‐regenerative effects of SPMs support the resolution of both cardiac and systemic inflammation.

**FIGURE 5:**
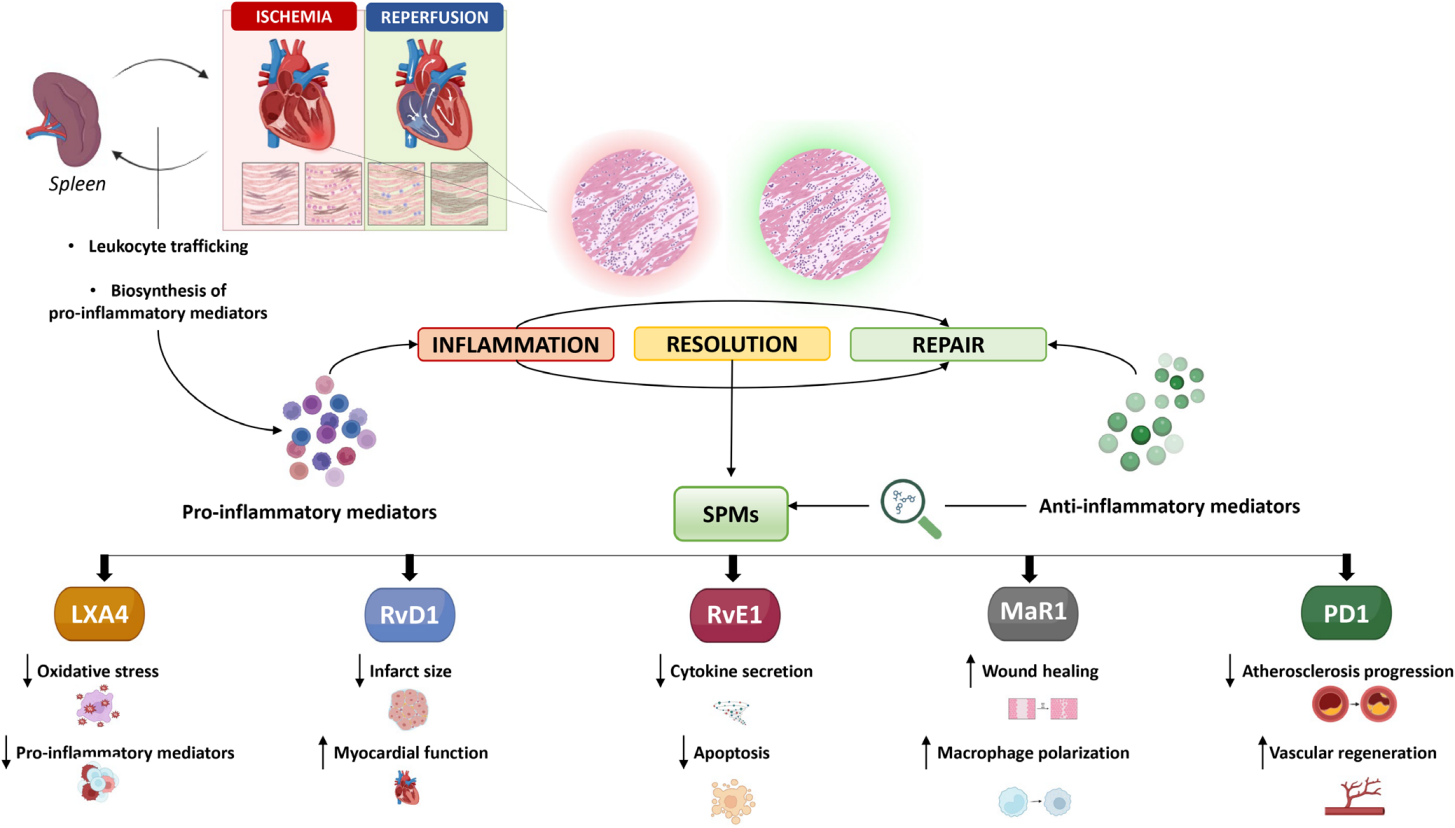
Specialized pro‐resolving mediators (SPMs) as key emerging players of the inflammatory resolution process in myocardial ischemia/reperfusion injury (MIRI). The figure highlights the potential cardioprotective role of SPMs, including Lipoxin A4 (LXA4), Resolvin D1 (RvD1), Resolvin E1 (RvE1), Maresin 1 (MaR1), and Protectin D1 (PD1), in modulating inflammation and facilitating cardiac repair following acute myocardial infarction (AMI). By reducing pro‐inflammatory mediator production and enhancing the clearance of cellular debris, SPMs contribute to the resolution of inflammation, ultimately conferring protection against MI and MIRI. Furthermore, the figure emphasizes the complex and dynamic spleen–heart cross‐talk, which orchestrates leukocyte trafficking and SPM biosynthesis in both the infarcted myocardium and the spleen after MI, ultimately shaping the delicate balance between inflammatory and pro‐resolving responses.

While experimental and clinical data highlight the critical role of SPMs in the heart and vasculature across various CVD, further research is necessary to fully elucidate the mechanisms underlying their beneficial effects in the initiation and resolution of MIRI. This includes identifying specific receptors and downstream signaling pathways that mediate their actions over both short‐ and long‐term periods. Figure 5 provides a comprehensive overview of the potential cardioprotective role of SPMs, highlighting those for which preclinical studies have demonstrated significant protective effects in MI and MIRI. Current preclinical evidence, along with a limited number of clinical studies on SPMs in the context of MIRI, suggests that promoting inflammation resolution by targeting SPM‐driven mechanisms, rather than merely suppressing inflammation, could be a promising therapeutic approach to mitigate ischemic tissue damage and prevent HF. This is particularly relevant for MIRI, where inflammation plays a central role in driving ventricular dysfunction following reperfusion injury, and an imbalance between pro‐ and anti‐inflammatory mediators is closely linked to progressive myocardial function decline.

## Author Contributions

**Anna De Bartolo:** methodology, data curation, writing – original draft. **Naomi Romeo:** writing – original draft, methodology, data curation. **Tommaso Angelone:** writing – review and editing, funding acquisition, supervision, data curation, conceptualization. **Carmine Rocca:** conceptualization, writing – review and editing, data curation, supervision, writing – original draft, methodology.

## Conflicts of Interest

The authors declare no conflicts of interest.

## Data Availability

Data sharing is not applicable to this article as no new data were created or analyzed in this study.
